# Influence of cellular shape on sliding behavior of ciliates

**DOI:** 10.1080/19420889.2018.1506666

**Published:** 2018-08-15

**Authors:** Yukinori Nishigami, Takuya Ohmura, Atsushi Taniguchi, Shigenori Nonaka, Junichi Manabe, Takuji Ishikawa, Masatoshi Ichikawa

**Affiliations:** a Department of Physics, Kyoto University, Sakyo, Kyoto, Japan; b Laboratory for Spatiotemporal Regulations, National Institute for Basic Biology, Okazaki, Japan; c Graduate School of Engineering, Tohoku University, Aoba, Sendai, Japan

**Keywords:** Sliding on the wall, ciliates, *Paramecium caudatum*, unicellular behaviors

## Abstract

Some types of ciliates accumulate on solid/fluid interfaces. This behavior is advantageous to survival in nature due to the presence of sufficient nutrition and stable environments. Recently, the accumulating mechanisms of *Tetrahymena pyriformis* at the interface were investigated. The synergy of the ellipsoidal shape of the cell body and the mechanosensing feature of the cilia allow for cells to slide on interfaces, and the sliding behavior leads to cell accumulation on the interfaces. Here, to examine the generality of the sliding behavior of ciliates, we characterized the behavior of *Paramecium caudatum*, which is a commonly studied ciliate. Our experimental and numerical results confirmed that *P. caudatum* also slid on the solid/fluid interface by using the same mechanism as *T. pyriformis*. In addition, we evaluated the effects of cellular ellipticity on their behaviors near the wall with a phase diagram produced via numerical simulation.

Ciliates, which belong to a group of protists, play crucial roles in ecosystems [1,2]. One of the characteristic features of ciliates is the presence of cilia on the entire cell surface, whose beating motions induce thrust force to swim in water. Ciliates exhibit remarkable behaviors using cilia, such as helical swimming in bulk water [3–5] and accumulation on air/fluid and solid/fluid interfaces [6,7]. Since nutrition is deposited on the interfaces, it is advantageous that ciliates prefer the interfaces. Recently, Ohmura *et al*. [7] revealed the mechanisms of the accumulating behavior of *Tetrahymena pyriformis* at a solid/fluid interface, which was induced by the sliding behavior of *T. pyriformis* on the surface. The experiments and numerical results exhibited a cellular shape and a mechanosensing feature of cilia, where a cilium contacting the wall almost stopped beating, causing the sliding behavior of *T. pyriformis*. Although the authors also observed sliding behavior of *Paramecium caudatum*, commonalities and differences in sliding behavior have not been compared quantitatively. In this paper, we examined the effects of the ellipsoidal cellular shape on the sliding behavior of *P. caudatum* compared to *T. pyriformis*.

First, we measured the behaviors of *P. caudatum*. Although, in bulk water, *P. caudatum* swam helically, in the bottom region, cells slid on the glass (Figure 1(A). The glass plate had been coated with MPC, which prevented nonspecific interaction between the cell and the glass, as revealed in Ohmura *et al*. [7]. The transient dynamics from the bulk motion into sliding motion on the solid/fluid interface were directly yielded by side-view observation, where the cell was incident to the wall and slid after contacting the side wall (Figure 1(B)). The steady angle $\theta_{s}$ was observed through lateral observation as 8.9 ± 0.2° (mean ± SEM, n = 244) (Figure 2(A). The traveling speed in bulk water was 948.9 ± 30.0 μms^−1^ (mean ± SEM, n = 73), and the traveling speed on the glass was 81.9 ± 3.4 μms^−1^ (mean ± SEM, n = 512), (Figure 2(B), which was qualitatively consistent with the simulation results of Ohmura *et al*. [7] where the sliding speeds were slower than the swimming speeds in bulk water. To examine whether the mechanisms of the sliding behavior of *P. caudatum* were the same as those of *T. pyriformis*, the flow fields generated by ciliary beating were determined by using 1.0 µm silica beads as tracer particles. Figure 2(C) shows the flow field caused by *P. caudatum* near the glass surface as estimated by the PIV analysis. This result revealed that little flow was produced at the region sandwiched between the cell and the glass, which meant the cilia near the glass surface did not produce thrust force. The map of the flow speeds visually supports this result (Figure 2(D). Similar results were also observed for *T. pyriformis*, and a small amount of flow was elicited by stopping ciliary beating, which was a key factor for swimmers to slide on the solid/fluid interface [7]. Therefore, it is suggested that the cilia of *P. caudatum* near the wall behave in the same manner as in the sliding motion of *T. pyriformis*, i.e., ciliary beating is stalled by attaching to the solid surface, which creates asymmetrical thrust forces around the cell, and consequently the cell experiences nose-down torque during the sliding motion on the solid surface.

**Figure 1.**
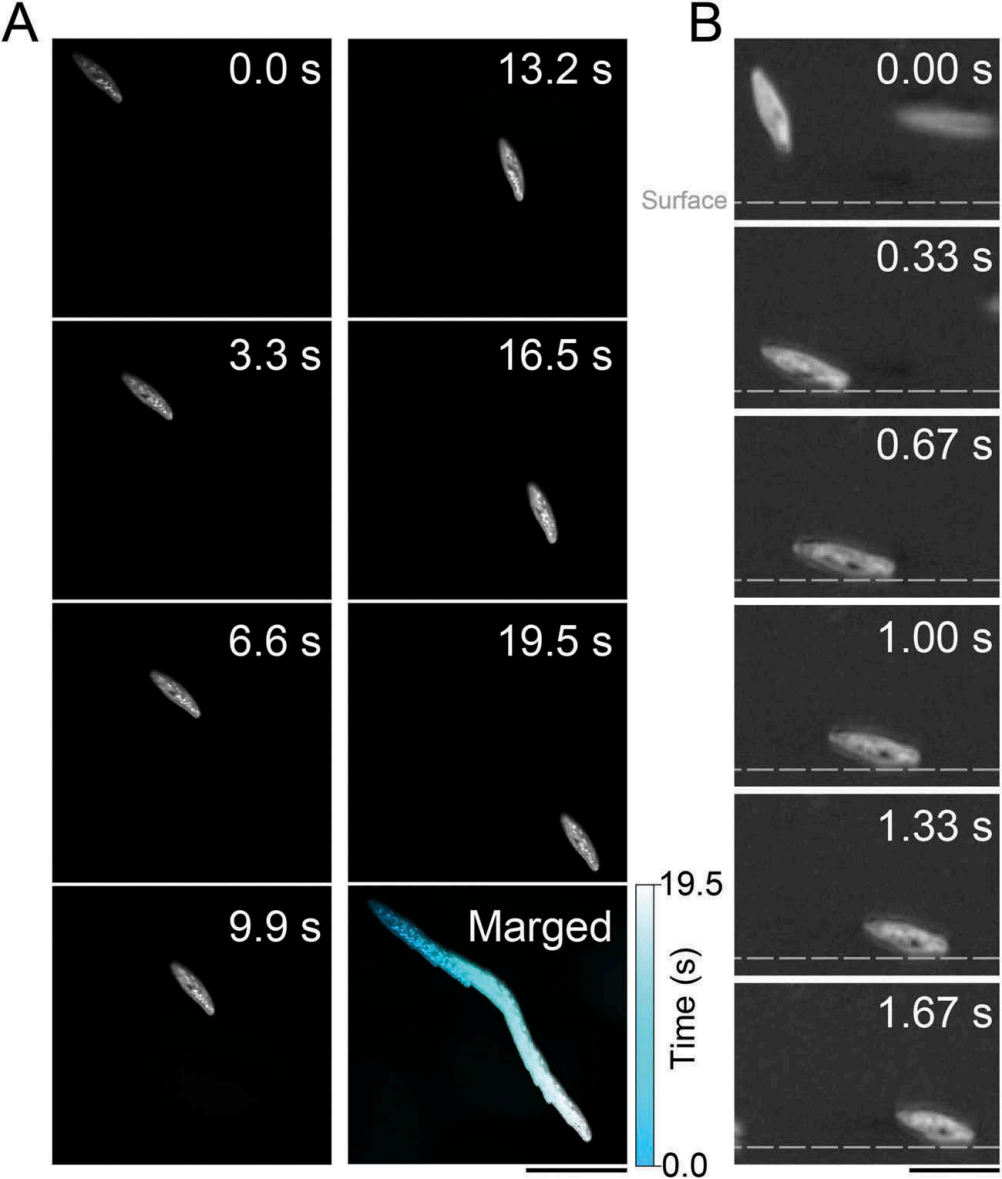
Sliding behavior of *Paramecium caudatum* on a glass surface. (A) A top view of *P. caudatum* sliding on a glass surface. Bar = 300 µm. (B) A side view of process of the sliding behavior of *P. caudatum* near the glass surface. Gray broken lines indicate glass surface. After touching the glass surface, the cell slid along the surface. Bar = 200 µm.

**Figure 2.**
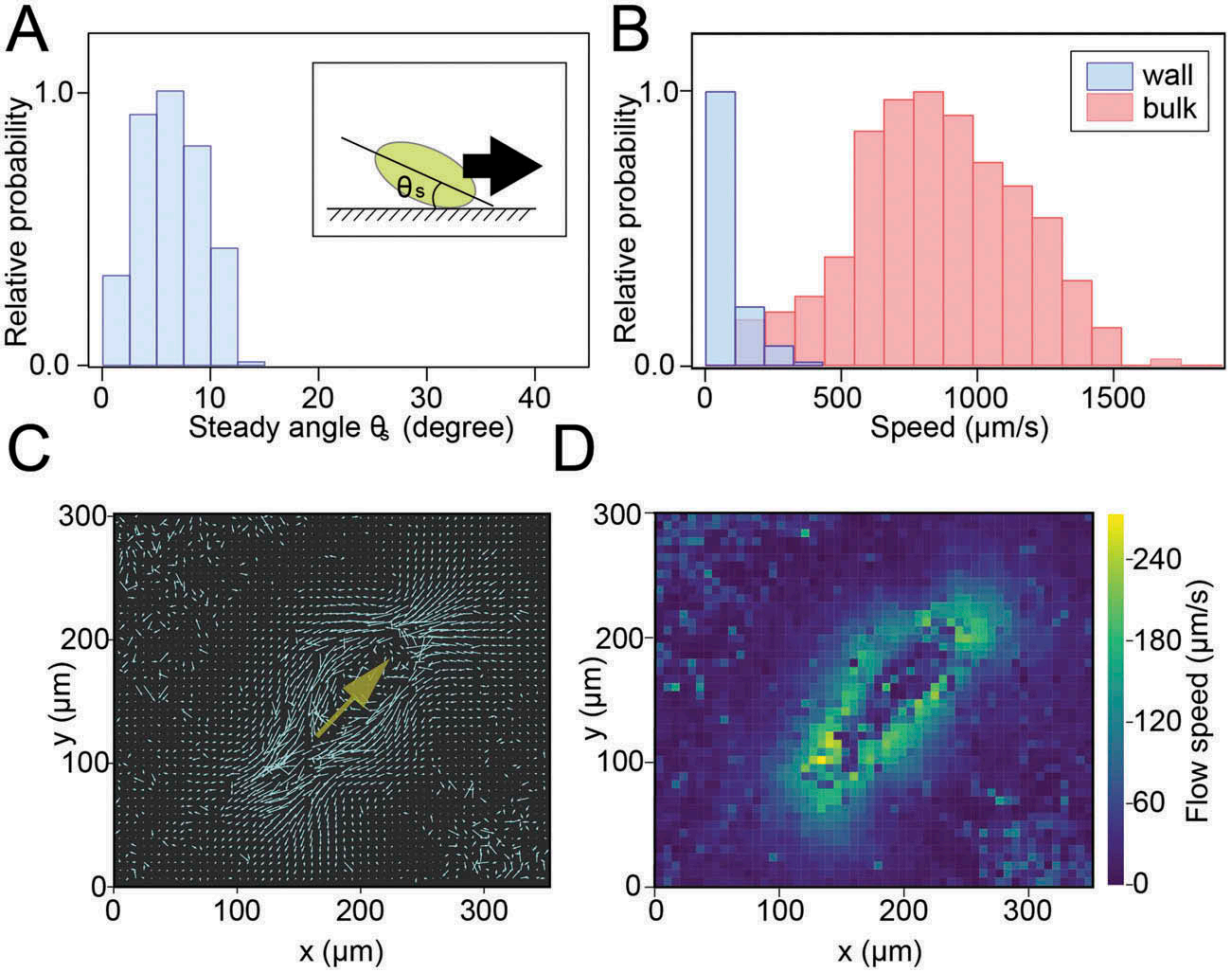
Analyses on the sliding behavior of *P. caudatum.* (A) Histogram of steady angles $\theta_{s}$ between the glass surface and longitudinal axis of the cell body (Inset). (B) Histogram of the swimming/sliding speeds of the cells in the bulk (red) and near the glass wall (blue). The speed near the glass surface was lower than in bulk water. (C) The flow field of *P. caudatum* near the glass surface was estimated by the PIV analysis. The yellow arrow indicates the swimming direction of the cell. The lateral side of the cell made rapid flows, and the bottom side produced slower flows. (D) Intensity map of flow velocity in (C).

In addition to the mechanosensing feature of cilia, Ohmura *et al*. [7] revealed that cellular shape was also an important factor to sliding on the surface. *P. caudatum*, however, has a more elongated shape than that of *T. pyriformis*. The aspect ratio of the cells $L_{1}/L_{2}$, where $L_{1}$ was the length of axis parallel to the ordinary swimming direction and $L_{2}$ was the length of axis perpendicular to the swimming direction, was 4.1 ± 0.1 (mean ± SEM, n = 11) in *P. caudatum*. This is approximately twice that of *T. pyriformis* [7]. To examine the influences of the aspect ratio of the cells, we performed hydrodynamic simulations using BEM (see supplemental online text). The simulations regarded the cell as a rigid ellipsoidal body with tangential thrust force above the body surface, and the feature by which cilia stalled their beating near a wall was represented by SBA, in which cilia did not produce thrust force (Figure 3(A). The wall was defined as a hydrodynamically non-slip and flat boundary. In the calculations, the variables were the SBA range $L_{SBA}$ and the aspect ratio $L_{1}/L_{2}$. Both $L_{SBA}$ and $L_{1}/L_{2}$ affect the area stopping ciliary beating. Of course, since the real cell shapes are more complex, the SBA should be treated as a qualitative and effective value. For the shape parameter, $L_{1}/L_{2}>1$ means prolate ellipsoidal, $L_{1}/L_{2}=1$ is spherical, and $L_{1}/L_{2}<1$ is oblate ellipsoidal. We changed the axis length $L_{1}$ parallel to the swimming direction and fixed the perpendicular length $L_{2}=1.0$. The initial distance between the wall and the center of mass of the swimmer was $h_{0}=4.0$, the initial swimming angle was $\theta_{0}=60.0$ and the time step was $\Delta t=1.0\times 10^{-3}$. The speed of the numerical swimmer was defined as U=1.0 in bulk water. In the initial setups, swimmers were placed near the wall and traveled in the direction of the wall. To compare with the steady sliding angle, the terminal angle $\theta_{t}$ after $3.0\times 10^{4}$ steps was yielded, which was enough time for swimming angles to converge in the present setup. In this coordinate, $\theta_{t}<0$ meant the swimmer left the wall (Figure 3(B) top), $\theta_{t}=90.0$ meant the swimmer swam vertically to the wall and seemed to stop on the wall (Figure 3(B) bottom), and $\theta_{t}>0$ meant the swimmer moved away from the wall (Figure 3(B) middle); the calculated $\theta_{t}$ was equivalent to $\theta_{s}$ in experiments. The simulations resulted in three behaviors as shown in the phase diagram (Figure 3(C). In the diagram, stopping motion was defined as when $\theta_{t}>88.5^{\circ }$ and swimming speed was less than 0.015 after $3.0\times 10^{4}$ steps, sliding behavior was defined as when $0^{\circ }<\theta_{t}<37.5^{\circ }$ and the swimming speed was more than 0.31, and leaving behavior was defined as when $\theta_{t}<0^{\circ }$. In the case of $L_{1}/L_{2}\;≦\;1,$ except for $L_{SBA}=\;0$ and $L_{1}/L_{2}=1$, swimmers exhibited stopping motion, and in others, shorter $L_{SBA}$ resulted in the leaving behavior, and longer $L_{SBA}$ induced the sliding behavior (Figure 3(C). In *P. caudatum*, $L_{SBA}=\;0.37\pm 0.08$ (mean ± SEM, n = 5) and $L_{1}/L_{2}=4.1\pm 0.1$ (mean ± SEM, n = 11) were estimated from experimental data: in *T. pyriformis*, $L_{SBA}=\;0.3$ and $L_{1}/L_{2}=2$ [7]; these parameters were within the region of the sliding motion. In addition, the aspect ratios and terminal angles of the sliding behaviors have negative correlations (Figure 3(D), consistent with the fact that the steady angle of *P. caudatum* was smaller than that of *T. pyriformis*.

**Figure 3.**
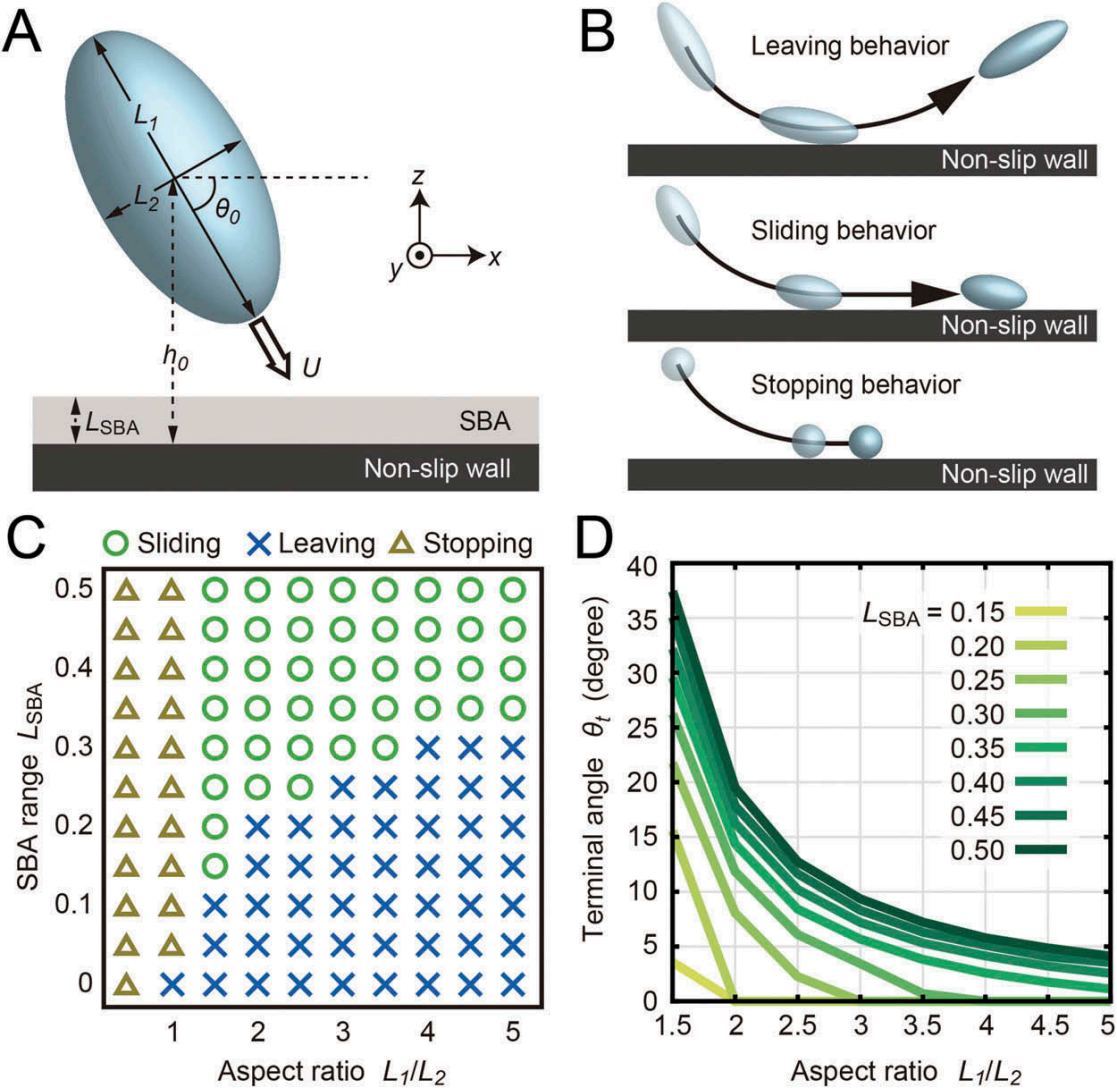
Schematic illustrations and results of the numerical calculations. (A) The cells were regarded as rigid ellipsoids with tangential thrust force above the body surfaces. We defined the SBA as the gray area on the bottom wall, where the thrust force vanished. The range of SBA was defined by $L_{SBA}$. The shape of the swimmer was parameterized by the ratio of $L_{1}$and $L_{2}$. The initial distance between the wall and the center of mass of the swimmer was $h_{0}=4.0$, and the initial swimming angle was $\theta_{0}=60.0$. (B) The behaviors of the swimmers were categorized into three motions: leaving (top), sliding (middle) and stopping (bottom) behaviors. (C) The phase diagram of the near-wall behavior of swimmers. The three behaviors were exhibited within the calculated ranges. The parameters of *P. caudatum* and *T. pyriformis* were within the parameters that induced the sliding behavior. (D) The aspect ratios and the terminal angles of the sliding behaviors had negative correlations.

Our experimental and numerical results suggested that *P. caudatum* also slid on the solid/fluid interface via the same mechanism as *T. pyriformis*, and the cell shape determined the behavior of the cells. Although the present experiment exhibited a point-to-point tendency in a large variety of ciliates, this simple result should contribute to considerations of an optimization strategy of ciliates for survival in nature.

## Methods

### Preparation of cells

*Paramecium caudatum* (Ai253) was purchased from the Symbiosis Laboratory, Yamaguchi University, with support from the National Bio-Resource Project (NBRP) of the Japan Agency for Medical Research and Development (NBRP-Paramecium, http://nbrpcms.nig.ac.jp/paramecium/). The cells were cultured in a barley grass solution (0.3 g/ml Young barley grass power (Yamamoto kanpoh Pharmacetical) dissolved in distilled water) bacterized with *Enterobacter aerogenes* at 25°C. Before observation, the cells in mid-log phase were washed three times with an observation solution (1.4 mM Na_2_HPO_4_, 0.6 mM NaH_2_PO_4_, 2 mM NaC_6_H_5_O_7_ citrate, and 1.5 mM CaCl_2_).

### Observations of cells

To prevent nonspecific binding between the cells and glass during observations, the glass (thickness no. 1, 30 × 40 mm; Matsunami) was coated with MPC polymer (Lipidure-CM5206; NOF Corporation) according to Ohmura et al. [7]. Briefly, MPC polymer was dissolved in ethanol to a final concentration of 1.0% (wt/vol), and 40 μL of MPC polymer solution was placed on the glass. To dry the coating solution, the glass was left at room temperature for more than 2 h. The observation solution containing *P. caudatum* was deposited between MPC-coated cover glasses with a spacer [a silicone sheet (thickness of 400 µm), which had a square hole (side length of 10 mm)]. *P. caudatum* was observed and recorded using a dark-field inverted microscope (Eclipse Ti; Nikon) with an sCMOS camera (ORCA-Flash4.0; Hamamatsu). To observe side views of *P. caudatum*, a smaller cover glass (thickness no. 1, 10 × 12 mm; Matsunami) was placed in a disposal cuvette with observation solution containing *P. caudatum*, and the interface between the glass and the observation solution was observed from an angle parallel to the ground using a free-angle observation system (VH-S30, VW-9000, VH-Z50L, and VW-600C; Keyence).

### Analysis and numerical methods

The cellular speeds and steady angles were acquired using Fiji (https://fiji.sc/), and the calculation of statistical analyses were performed using Igor Pro 6.37 (WaveMetrics). In the PIV analysis, Python (https://www.python.org/) with OpenPIV (http://www.openpiv.net/) was used. Numerical calculation was performed according to Ohmura et al. [7]. The detailed method is described in Supplemental online text.

## Abbreviations

MPC, 2-methacryloyloxyethyl phosphorylcholine; PIV, particle image velocimetry; BEM, boundary element method; SBA, stop beating area
